# *Pueraria mirifica* Exerts Estrogenic Effects in the Mammary Gland and Uterus and Promotes Mammary Carcinogenesis in Donryu Rats

**DOI:** 10.3390/toxins8110275

**Published:** 2016-11-04

**Authors:** Anna Kakehashi, Midori Yoshida, Yoshiyuki Tago, Naomi Ishii, Takahiro Okuno, Min Gi, Hideki Wanibuchi

**Affiliations:** 1 Department of Molecular Pathology, Osaka City University Graduate School of Medicine, 1-4-3 Asahi-machi, Abeno-ku, Osaka 545-8585, Japan; Yoshiyuki_Tago@kn.kaneka.co.jp (Y.T.); naomi-u@med.osaka-cu.ac.jp (N.I.); m2026860@med.osaka-cu.ac.jp (T.O.); mwei@med.osaka-cu.ac.jp (M.G.); wani@med.osaka-cu.ac.jp (H.W.); 2 Division of Pathology, Biological Safety Research Center, National Institute of Health Sciences, Ministry of Health, Labour and Welfare, 1-18-1 Kamiyoga, Setagaya-ku, Tokyo 158-8501, Japan

**Keywords:** *Pueraria mirifica*, mammary gland, uterus, carcinogenesis, estrogenic activity, Donryu rat

## Abstract

*Pueraria mirifica* (PM), a plant whose dried and powdered tuberous roots are now widely used in rejuvenating preparations to promote youthfulness in both men and women, may have major estrogenic influence. In this study, we investigated modifying effects of PM at various doses on mammary and endometrial carcinogenesis in female Donryu rats. Firstly, PM administered to ovariectomized animals at doses of 0.03%, 0.3%, and 3% in a phytoestrogen-low diet for 2 weeks caused significant increase in uterus weight. Secondly, a 4 week PM application to non-operated rats at a dose of 3% after 7,12-dimethylbenz[a]anthracene (DMBA) initiation resulted in significant elevation of cell proliferation in the mammary glands. In a third experiment, postpubertal administration of 0.3% (200 mg/kg body weight (b.w.)/day) PM to 5-week-old non-operated animals for 36 weeks following initiation of mammary and endometrial carcinogenesis with DMBA and *N*-ethyl-*N*′-nitro-*N*-nitrosoguanidine (ENNG), respectively, resulted in significant increase of mammary adenocarcinoma incidence. A significant increase of endometrial atypical hyperplasia multiplicity was also observed. Furthermore, PM at doses of 0.3%, and more pronouncedly, at 1% induced dilatation, hemorrhage and inflammation of the uterine wall. In conclusion, postpubertal long-term PM administration to Donryu rats exerts estrogenic effects in the mammary gland and uterus, and at a dose of 200 mg/kg b.w./day was found to promote mammary carcinogenesis initiated by DMBA.

## 1. Introduction

*Pueraria mirifica* (PM), also known as white Kwao Krua, is a plant found in northern and northeastern Thailand which belongs to the family of Leguminosae, and the soy, bean, and pea subfamily Papilionoideae. Previously, PM application for treatment of a range of conditions, including those related to the aging process, has been reported [1]. Dried and powdered tuberous roots of PM contain at least 17 chemical compounds with estrogenic biological activities, usually divided into three groups: the first group includes teniso flavonoids such as genistin, genistein, daidzein, daidzin, kwakhurin, kwakhurin hydrate, tuberosin, puerarin, mirificin, and puemiricarpene [2,3]; the second group of coumestans comprises coumestrol, mirificoumestan, mirificoumestan glycol, and mirificoumestan hydrate; and the third features chromenes, such as miroestrol, deoxymiroestrol, and isomiroestrol [2]. All these substances are phytoestrogens with structures similar to that of 17 β-estradiol. Miroestrol, the phytoestrogen with the highest estrogenic activity among all those isolated from PM, is considered similar to estriol, which is considered the safest estrogen for humans [4,5,6,7]. Furthermore, PM has been reported to contain phytoestrogens like β-sitosterol, stigmasterol, campesterol, as well as the cytotoxic non-phytoestrogen spinasterol [3,8]. Puerarin might account for about half of the total isoflavone content of PM, with lower amounts of genistin and daidzin present, all these being glycoside forms which can be partially hydrolyzed in the intestine by C-glycosyl bond cleavage to give the respective aglycoside forms: genistein, daidzein, and daidzein [2].

Nowadays, PM is available in tablets, extracts, creams, sprays, and powdered forms, so that it can be added to other medicinal preparations or herbs, and individual conditions require different applications and dosages [1]. It can be readily obtained from internet resources in many countries, including the USA and Japan, and is primarily used for supporting memory, smoothing the skin, increasing hair growth, improving appetite, and providing relief for ailments like osteoporosis and even cancer [9,10,11,12]. Continuous administration of PM at 20–100 mg/day for 6 months, or at 100–200 mg/day for 12 months, was found to help women having menopause symptoms, while no significant changes were detected in their hepatic, hematologic, and renal functions [3]. Pretreatment with PM at a high dose (1000 mg/kg body weight (b.w.)/day) for 4 weeks was shown to suppress the development of mammary tumors induced by 7,12-dimethylbenz[a]anthracene (DMBA) in Sprague–Dawley rats [13]. However, the effect of long-term administration at different doses has not yet been clarified in detail. Based on available studies, a safe dosage of PM as a dietary supplement for humans was suggested at 1–2 mg/kg b.w./day or about 50–100 mg/day [3]. Nowadays, doses of 20–100 mg/day are commonly used, but in some cases 200–900 mg/day or even higher (up to 3000 mg/day) are applied. Until now, no serious side effects have been recorded with the prescribed safe dosage, although at high doses PM may cause epilepsy, diabetes, asthma, and migraine [3].

Despite the data on the benefits of PM, there are reasons for concern that a herb which exhibits strong estrogen-like properties may stimulate the growth of existing estrogen-sensitive breast or endometrial tumors, pointing out questions such as: what is a safe dose? Previously, nanomolar concentrations of genistein, present in PM, was shown to induce acid ceramidase (ASAH1) transcription through a GPR30-dependent, pertussis toxin-sensitive pathway that requires the activation of c-Src and extracellular signal regulated kinase 1/2 (ERK1/2), thus stimulating breast cancer cell growth [14]. Recently, we further demonstrated that postpubertal administration of soy isoflavones at estrogenic doses promotes mammary and endometrial carcinogenesis in Donryu rats [15]. These data call into question the safety of long-term exposure to phytoestrogens with regard to effects on the mammary gland and endometrium. It is of particular importance that concentrations of PM which might exert promoting effects on mammary gland and uterine carcinogenesis be determined. Therefore, the present study was carried out to investigate the modifying effects of various doses of PM on mammary and uterine endometrial carcinogenesis using the Donryu rat model.

## 2. Results

### 2.1. Estrogenic Effect of Test Compounds (Short-Term Experiment 1)

**Figure 1:**
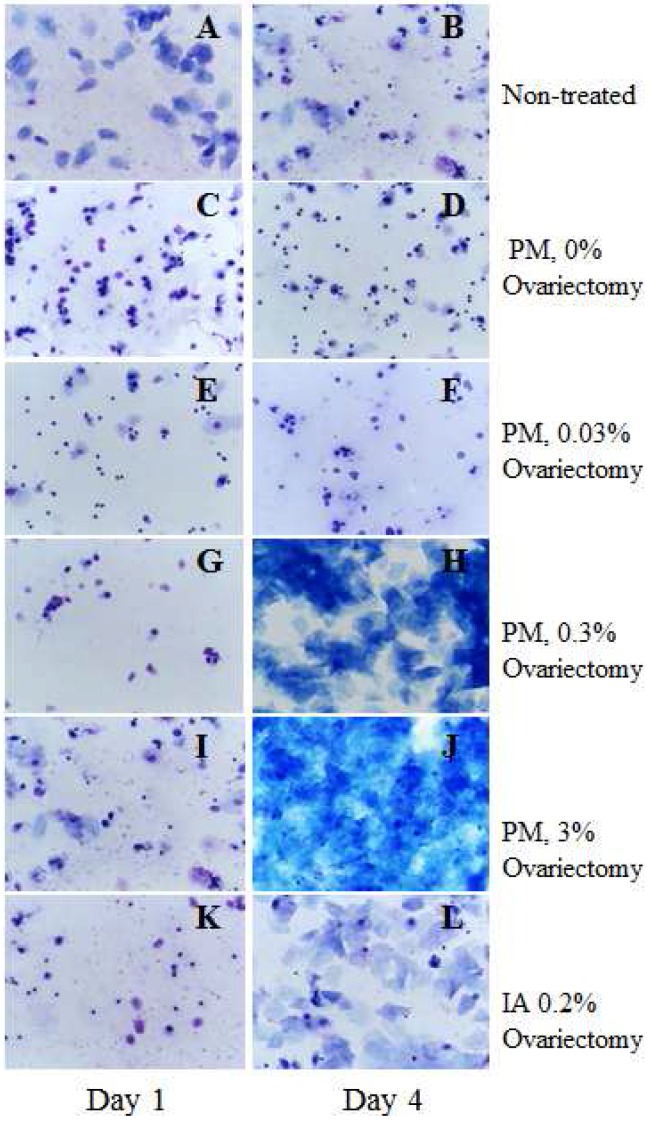
Vaginal cytology for non-treated (**A**,**B**) and ovariectomized (**C**–**L**) female Donryu rats administered *Pueraria mirifica* (PM) and isoflavone aglycon (IA) for the first 4 days. Animals were given PM at doses of 0 (**C**,**D**), 0.03 (**E**,**F**), 0.3 (**G**,**H**) and 3% (**I**,**J**), or 0.2% IA (**K**,**L**) 2 weeks after the ovariectomy. Vaginal smears were obtained daily before and after starting the treatment, dried and stained with an aqueous solution of methylene blue. In the ovariectomized rats the absence of cyclicity was confirmed by castration smears typical of diestrus. Note that 0.3% and 3% PM as well as 0.2% IA rats exerted estrogenic activities confirmed by cornification which was similar to estrus status.

After ovariectomy, PM treatment at doses of 0.3% and 3% and isoflavone aglycon (IA) treatment at a dose of 0.2% resulted in cornification, evident on examination of vaginal smears (Figure 1).

The 0.3% PM treatment was found to exert even stronger estrogenic activity than 0.2% IA. Mean relative uterus weights were significantly elevated in 0.03% (0.13% ± 0.01%, *p* < 0.05), 0.3% (0.31% ± 0.03%, *p* < 0.05), 3% PM (0.35% ± 0.08%, *p* < 0.05) and 0.2% IA (0.19% ± 0.04%, *p* < 0.05) administered ovariectomized rats as compared to the control (0.08% ± 0.01%). Thus, weak, medium, and strong estrogenic activities of PM at doses of 0.03% (20 mg/kg b.w./day), 0.3% (200 mg/kg b.w./day), and 3% (2000 mg/kg b.w./day), respectively, were demonstrated in the rat uterus. In this experiment, significant decreases of body weights were observed in the 0.3% and 3% PM groups (*p* < 0.001) as well as 0.2% IA group (*p* < 0.01). Because of the very strong estrogenic effect of 3% PM detected in the short-term study, in the succeeding long-term experiment the highest dose was changed from 3% to 1%. Thus, the test doses of PM in experiment 3 were set as: low, 0.03%; medium, 0.3%; and high, 1%.

### 2.2. Cell Proliferation in the Mammary Gland (Short-Term Experiment 2)

**Figure 2:**
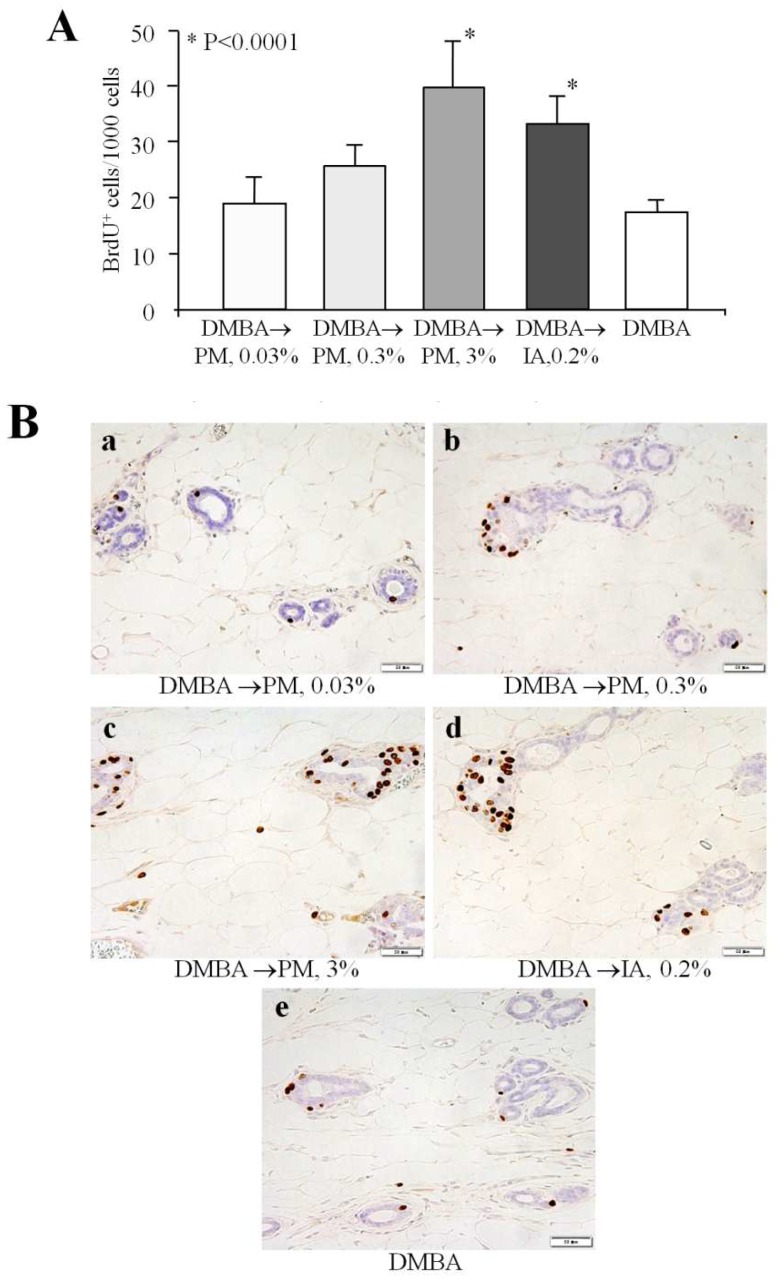
Bromodeoxyuridine (BrdU) labeling indices in the mammary glands of rats administered PM and IA after the 7,12-dimethylbenz[a]anthracene (DMBA) initiation (**A**); representative pictures of BrdU immunohistochemistry of mammary glands of rats (**B**). Note the dose-dependent induction of cell proliferation by the short-term application of PM as compared to the DMBA initiation control rats.

Bromodeoxyuridine (BrdU) immunohistochemistry revealed a dose-dependent increase of cell proliferation in the terminal end buds of mammary glands of PM-treated rats after 4 weeks of administration (Figure 2). Significant elevation of the number of BrdU positively (BrdU^+^)-stained cells was noted in 3% PM (*p* < 0.001) and 0.2% IA groups (*p* < 0.001), as compared to the DMBA control group.

### 2.3. Long-Term Study (Experiment 3)

#### 2.3.1. Body, Organ Weights, Food, and Water Consumption

**Figure 3:**
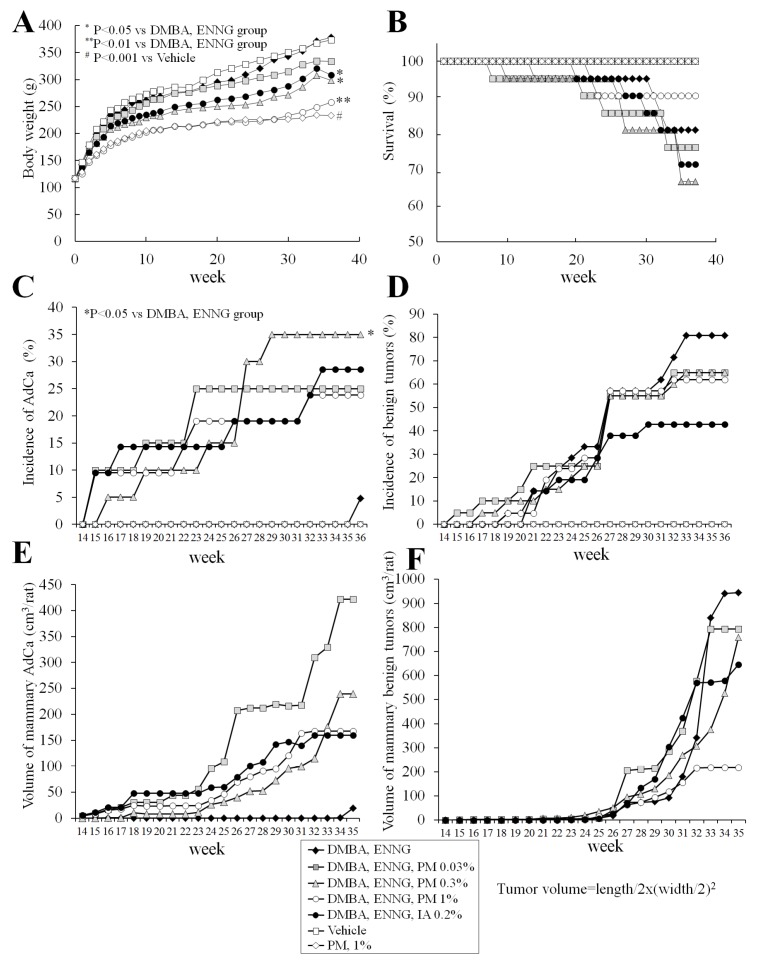
Body weight (**A**) and survival (**B**) curves of female Donryu rats in experiment 3; incidences of mammary adenocarcinomas (**C**), benign tumors (**D**), and volumes of mammary adenocarcinomas (**E**) and benign tumors (**F**). Note the significant decreases of body weights in the 0.3 and 1% PM- and 0.2% IA-treated Donryu rats. Trends for decrease in survival were found for 0.03 and 0.3% PM and 0.2% IA groups. Significant increases of mammary adenocarcinoma incidence were observed in 0.3% PM- and 0.2% IA-treated rats. Adenocarcinomas in PM- and IA-treated rats appeared earlier, and their volumes were higher than in the initiation control group. Development of benign tumors in the initiation control group starting at week 32 was evident. Mammary tumors were larger in animals with higher body weights.

Rat body weight curves are presented in Figure 3A. Body weights of 0.3% PM-, 1% PM-, and 0.2% IA-administered rats were lower than in the initiation control group, with significant differences detected at the termination of the experiment. No variation in food intake was observed among groups but decreased water consumption was noted in PM- and IA-treated animals.

Significant increases of relative uterus weight were found with 0.3 and 1% PM, and a trend for increase was found in the vehicle 1% PM group, as compared to the respective control groups (Supplementary Materials Table S1). Relative liver weights were significantly decreased in 0.3 and 1% PM-treated rats. Moreover, significant elevation of relative kidney weights was found in the 1% PM group. In addition, significant increase of relative thymus weights at 0.03% PM and elevation of relative adrenal weights in the vehicle 1% PM group as compared to the respective controls were noted.

#### 2.3.2. Survival

Changes in rat survival are shown in Figure 3B. Four animals in the initiation control, 6 rats in the 0.03% PM, 7 rats in the 0.3% PM, 2 rats in the 1% PM, and 6 rats in the 0.2% IA groups died during the study. One rat each in the vehicle control, 0.03% PM, and 0.3% PM groups were found dead with no discernible cause. The apparent causes of death in the initiation control group were zymbal gland tumor (1 rat) and malignant lymphoma/leukemia (3 rats). In contrast, causes of death in the 0.03%, 0.3%, 1% PM and 0.2% IA groups were mammary adenocarcinomas (0.03% PM, 2 rats; 0.3% PM, 2 rats; 1% PM, 1 rat; IA, 3 rats), with bleeding from large necrotic mammary tumors (0.03% PM, 2 rats; 0.3% PM, 1 rat; 1% PM, 1 rat), lymphoma/leukemia (0.03% PM, 2 rats; 0.3% PM, 1 rat; IA, 2 rats), thymoma (0.3% PM, 1 rat), and uterine carcinoma (IA, 1 rat). Two rats in the 0.03% PM group, 1 rat receiving 0.3% PM, and 3 animals in the IA-treated group featured metastasis from mammary adenocarcinomas in the lung.

The first rat was found dead at week 9, from the 0.03% PM group, and the cause of death was lymphoma/leukemia. Subsequently, one rat in the 0.3% PM group at week 10 and one rat from the initiation control group at week 15 died from lymphoma/leukemia. The numbers of animals in the 0.03 and 0.3% PM- and IA-treated groups, but not the 1% PM group, then started to decrease mostly due to the development of mammary adenocarcinomas (Figure 3B). Survival rates of 0.03 and 0.3% PM- and IA-administered rats showed a nonsignificant trend for decrease at the termination of the experiment.

#### 2.3.3. Histopathological Analysis of Mammary Glands

**Figure 4:**
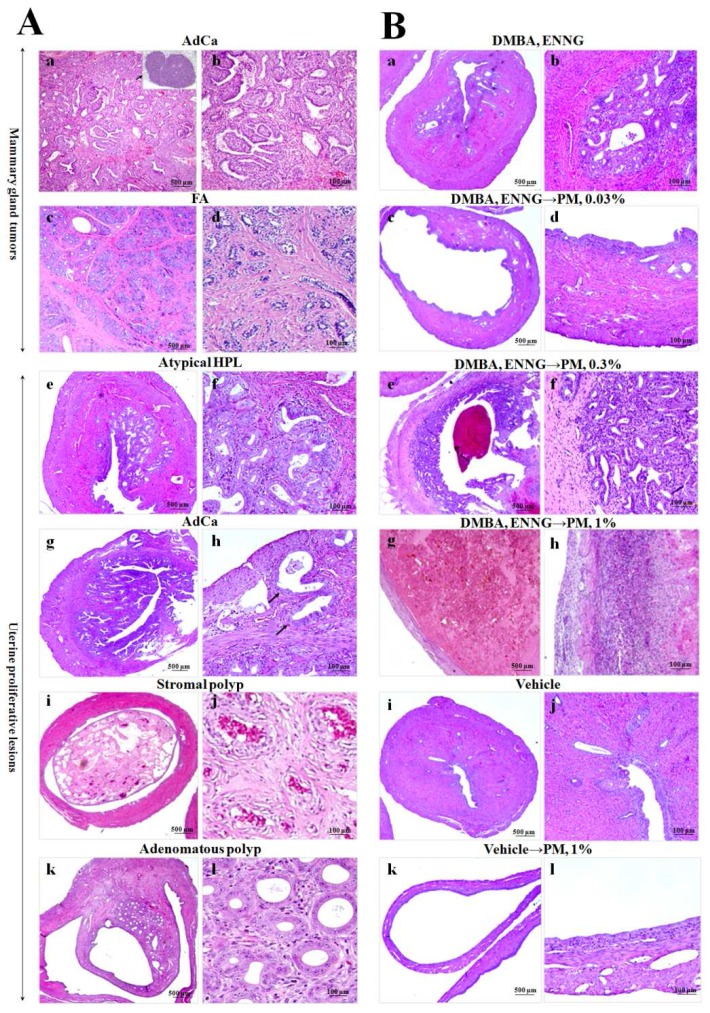
(**A**) Histopathological changes observed in mammary glands (**a**–**d**) and uteri (**e**–**l**) of initiation control, PM-, and IA-administered rats in experiment 3 (hematoxylin and eosin). Moderate atypical hyperplasia featuring an increased number of glands under the lining epithelium (**e**,**f**). Well-differentiated endometrial adenocarcinoma (AdCa) (**g**,**h**). Atypical glands present in the endometrium proliferating irregularly and invading the muscle layer (arrows). Stromal polyp (**i**,**g**) and adenomatous polyp (**k**,**l**); (**B**) histopathological changes in the uteri of the DMBA, *N*-ethyl-*N*′-nitro-*N*-nitrosoguanidine (ENNG) control (**a**,**b**), 0.03 (**c**,**d**), 0.3 (**e**,**f**), and 1% (**g**,**h**) PM-treated Donryu rats after initiation; vehicle control (**i**,**j**) and vehicle 1% PM (**k**,**l**)-administered rats in experiment 3. Note the development of atypical hyperplasia (DMBA, ENNG → 0.3% PM group), uterus dilatation (0.3 and 1% PM-treated rats), inflammation and hemorrhage (DMBA, ENNG → 0.3 and 1% PM groups) induced by the PM treatment. (Magnifications in (**A**) and (**B**): ×20 (**a**,**c**,**e**,**g**,**i**,**k**) and ×200 (**b**,**d**,**f**,**h**,**j**,**l**)).

Data for changes in mammary gland adenocarcinoma and benign tumor incidences, multiplicities, and volumes—with representative pictures—are shown in Figure 3C–F, Figure 4A(c,d) and Table 1.

Results of histopathological analysis demonstrated significant increase of incidence (*p* < 0.05) and the strong trend for increase of multiplicity of mammary adenocarcinomas in 0.3% PM- (*p* = 0.05) and IA-administered rats (Figure 3C and Table 1). There was a significant negative linear trend for multiplicity of fibroadenoma in PM-treated rats, and significant inhibition in IA-treated rats (*p* < 0.05) (Figure 3D and Table 1).

Macroscopically measured mammary adenocarcinoma volumes were elevated in the 0.03, 0.3, and 1% PM and 0.2% IA groups starting from week 16 as compared to the initiation control rats, with the highest value observed in the 0.03% PM dose group and the lowest increase induced by PM at a dose of 1% (Figure 3E). Unexpectedly, sudden development of benign tumors in the mammary glands of the initiation control rats was observed at weeks 32–36. Animals with higher body weights had larger mammary tumors (Figure 3F).

#### 2.3.4. Histopathological Analysis of Uteri

Data from histopathological examination of rat uteri are shown in Table 1 and Figure 4A(e–l),B(a–l). At termination, the uteri of 0.3 and 1% PM- as well as IA-treated rats after initiation of endometrial carcinogenesis demonstrated dilatation, increased hemorrhage, and higher numbers of nodules macroscopically. In the uteri of *N*-ethyl-*N*′-nitro-*N*-nitrosoguanidine (ENNG)-initiated rats, we observed various proliferative lesions, with a sequence of changes from atypical hyperplasias to adenocarcinomas (Figure 4A(e–h). In addition, stromal and adenomatous polyps were apparent (Figure 4A(i–l). A significant increase in the multiplicity of total atypical hyperplasias (HPLs) (mild, medium, and severe) was found in 0.3% PM- and IA-administered rats (Table 1). Furthermore, significant elevation of multiplicity of mild atypical HPLs was detected in the uteri of 0.03 and 0.3% PM groups. In addition, multiplicities of stromal and adenomatous polyps tended to increase with the 0.3% and 1% PM treatment, while significant elevation was detected in the uteri of the IA-administered rats (Table 1).

#### 2.3.5. Blood Hematology and Biochemistry

The results of hematological and biochemical examinations of the blood are presented in Supplementary Materials Table S2.

Significantly decreased red blood cell count, Hb, and Ht, but increased platelet count, were observed in DMBA- and ENNG-initiated rats as compared to the vehicle control rats. When PM was administered at a dose of 1% without initiation, Ht and lymphocyte count were significantly suppressed but neutrophils elevated, indicating higher levels of inflammation. Furthermore, PM administration after the initiation of mammary and uterine carcinogenesis significantly and dose-dependently suppressed the platelet counts.

In blood biochemistry, significant inhibition and a trend for decrease in total cholesterol and triglyceride levels were found in PM- and IA-administered rats after initiation and in the vehicle 1% PM group. Moreover, in 1% PM- and IA-treated rats, aspartate aminotransferase (AST) and alanine aminotransferase (ALT) were suppressed, whereas alkaline phosphatase (ALP) and γ-glutamyl transpeptidase (γ-GTP) were elevated. Furthermore, in the initiation control and 1% PM vehicle groups, the blood urea nitrogen (BUN) level was increased as compared to the vehicle control group. In addition, significant reduction and trends for decrease of blood calcium levels were found in 1% PM and other PM groups, respectively, as compared to the controls.

## 3. Discussion

The present results demonstrated that long-term postpubertal exposure to PM at doses higher than 200 mg/kg b.w./day exerts estrogenic activity and induces cell proliferation in the mammary glands of Donryu rats. Furthermore, long-term treatment with PM at 200 mg/kg b.w./day promoted mammary and endometrial carcinogenesis after DMBA and ENNG initiation. Mammary adenocarcinomas metastasizing to the lungs were found in 0.03 and 0.3% PM-treated rats, as well as 0.2% IA treated rats. In this study, the modifying effects of 0.3% PM on mammary gland and uterus were comparable with those of 0.2% IA. In the medium and especially in the high dose PM groups, decreases of rat body weights, adipose deposition, and total cholesterol and triglyceride levels in the blood were obvious, likely due to antilipogenic effects of estrogenic compounds described previously, or perhaps to the decrease of water intake of rats given PM [16]. In the long-term experiment, we observed significant increase of mammary adenocarcinoma development induced by PM at a dose of 0.3%. The absence of dose-dependence in the effects of PM on mammary adenocarcinomas may be related to side effects exerted at high doses, with best incorporation of its ingredients reaching working “physiological” intracellular concentrations at a dose of 0.3%.

Interestingly, short- and long-term administration of PM applied at medium and high doses resulted in increase of uterus weight, and dilatation, hemorrhage, and inflammation of the uterine wall. However, the influence of PM on lesion development in the uterus was much less pronounced as compared to that in the mammary gland—and only atypical hyperplasia was elevated—at 0.3% PM.

Trophic effects of estrogenic compounds on the mammary gland and uterus were previously suggested to be due to activation of signaling through estrogen receptors (ERs) ERα and ERβ [17]. It was reported that PM phytoestrogens at high doses could effectively outcompete 17 β-estradiol binding to ERα in MCF-7 cells [3]. We have previously shown that IA at an estrogenic dose of 150 mg/kg b.w./day activated ERα or ERβ and downstream AP1 and NF-κB transcriptional factors, also potentiating F-actin signaling in mammary and uterine adenocarcinomas [15]. However, the effects of biological substances possessing estrogenic activity generally appear to be dependent on the dose. In case of IA intake, “physiological” concentrations are known as those achieved in the serum of persons consuming commonly recommended daily doses of isoflavones of 50–100 mg [18]. However, in the case of PM, there are almost no data concerning the concentration of ingredients in the blood and tissues. The safe dose for humans is considered as 1–2 mg/kg b.w./day. From our results, the dose of PM exerting promoting effects on mammary and uterine carcinogenesis in rats was close to 200 mg/kg b.w./day (0.3%).

In female monkeys, daily treatment with 100 mg and 1000 mg/day (about 20 and 200 mg/kg b.w./day) of PM for 90 days produced a dose-dependent reduction in the urinary follicular stimulating hormone (FSH), luteinizing hormone (LH) and estradiol levels in the blood, and the single dose of 1000 mg disturbed ovarian function and menstrual cycling [19,20,21,22]. Furthermore, recent experiments in mice demonstrated that oral exposure to a nontoxic PM dose of 100 mg/kg b.w./day for 8 weeks resulted in prolonged estrous cycles, while 10 mg/kg b.w./day did not induce any changes in the hypothalamic–pituitary–ovarian–uterine axis, and did not exert estrogenic activity or adverse effects on mating efficiency or reproduction [23]. In addition, development of uterine endometrial hyperplasia and a decrease in the number of growing ovarian follicles, possibly related to reduction in the luteinizing hormone (LH) and follicle stimulating hormone (FSH) levels, were detected after PM application to mice at a dose of 100 mg/kg b.w./day but not at 10 mg/kg b.w./day. Moreover, in studies with gonadectomized female rats, oral treatment with water-suspended PM at doses of 100 and 1000 mg/kg b.w./day for 2 weeks resulted in a significant increase of uterine weight, remarkable vaginal and uterine proliferation, vaginal cornification, and suppression of reproductive functions [24,25,26,27,28]. Recovery after cessation of treatment was dependent on the dosage of PM.

In line with the previous results in rats and mice, in our short-term study we observed estrogenic effects of PM applied to ovariectomized rats at doses of 200 and 2000 mg/kg b.w./day for 2 weeks. Long-term PM administration at a dose of 0.3% (200 mg/kg b.w./day) was found not only to exert estrogenic activity in the mammary gland and uterus, but also to promote mammary carcinogenesis and induce atypical hyperplasia in the uteri of Donryu rats.

It is important to further mention that the timing of exposure to substances with estrogenic bioactivities is thought to be critical for effects on breast cancer risk. Thus, prepubertal and postpubertal exposure to estrogenic compounds such as genistein could have different effects on cell proliferation in the terminal ductules of mammary glands [29]. In the present case, postpubertal exposure to PM, isoflavones or other test compounds with estrogenic activity induced cell proliferation and promoted mammary and uterine carcinogenesis in our two-step carcinogenesis model with DMBA and ENNG initiation.

We also observed that 1% PM suppressed lymphocyte and platelet counts but elevated neutrophil levels, presumably reflecting effects on the immune system, promoting inflammation, and bone marrow suppression. Moreover, 1% PM elevated the ALP and γ-GTP as well as the BUN in the blood, which could occur if kidneys or liver were damaged. In addition, the present results demonstrated that 1% PM induced a decrease in blood calcium levels. These data are in line with recent results demonstrating that long-term treatment of aged menopausal monkeys with 1000 mg/day of PM decreased serum parathyroid hormone (PTH) and calcium levels in the blood likely due to the amelioration of the bone loss caused by estrogen deficiency [11,12].

In conclusion, in the present study long-term postpubertal treatment of Donryu rats with PM at a dose of 200 mg/kg b.w./day exerted promoting effects on mammary carcinogenesis after the initiation with DMBA. Furthermore, PM elevated cell proliferation in the mammary glands of DMBA-initiated rats, which might lead to the promotion and progression of mammary tumors to greater malignancy. In addition, it inhibited the levels of calcium in the blood, and induced inflammation, hemorrhage, and dilatation of the uterine wall in rats.

## 4. Experimental Section

### 4.1. Chemicals

*N*-ethyl-*N*′-nitro-*N*-nitrosoguanidine (ENNG) was obtained from Nakalai Tesque (Kyoto, Japan), 7,12-dimethylbenz[a]anthracene (DMBA) from Tokyo Chemical Industry Co. Ltd. (Tokyo, Japan) and polyethylene glycol (PEG) from Wako Pharmaceutical, Osaka, Japan. Other chemicals were from Sigma Chemical Co. (St Louis, MO, USA) or Wako Pharmaceutical (Osaka, Japan).

### 4.2. Test Compounds

The *Pueraria mirifica* powder (Lot No.: PM490621) was produced by Seiko Yakuhin Kogyo K.K. (Narashino, Chiba, Japan) and consigned by Shiratori Pharmaceutical Co., Ltd., (Narashino, Chiba, Japan) and Pias Corporation (Osaka, Japan). The taxonomic and content identification was performed by the Seiko Yakuhin Kogyo K.K. The sample contained miroestrol (5.3 mg/kg; 0.00053%), deoxymiroestrol (6.3 mg/kg; 0.00063%), puerarin (6′-*O*-beta-apiofuranoside: 21.7 mg/kg; 0.00217%), daidzin (daidzein-7-*O*-glucoside: 12.9 mg/kg; 0.00129%), genistin (genistein-7-*O*-glucoside: 8.7 mg/kg; 0.00087%), daidzein (7,4′-dihydroxyisoflavone: 48.2 mg/kg; 0.00482%), genistein (25.5 mg/kg; 0.00255%) and kwakhurin (3-[2-(3,3-dimethylallyl)-4,6-dihydroxy-3-methoxyphenyll-7-hydroxyisoflavone: 3.5 mg/kg; 0.00035%).

Isoflavone aglycon (IA) extract (SoyAct) was from Kikkoman Corporation (Noda City, Chiba, Japan). In the present experiment, test powder diets were prepared as follows: 0.03%, 0.3%, 1, and 3% PM diets contained 0.03%, 0.3%, 1, and 3% *Pueraria mirifica* powder, respectively, in NIH-07PLD powder diet (Phytoestrogen Low Diet, Oriental Yeast, Tokyo, Japan). The accuracy of dose formulation and uniformity of blending of the diets was confirmed by the analytical chemistry laboratories at Oriental Yeast Co., Tokyo, Japan. For the production of IA extract fermentation of soy was performed followed by ethanol/water extraction and purification [15].

### 4.3. Animals

One hundred and seventeen female 4-week-old Crlj:DON (Donryu) rats (Japan SLC, Shizuoka, Japan) were obtained at 5 weeks of age and quarantined for 1 week before the experiment started. The rats were kept in an animal house with a 12 h (8:00–20:00) light/dark cycle, humidity of 50% ± 2% and temperature of 23 ± 1 °C. Tap water and NIH-07PLD diet (Phytoestrogen Low Diet, Oriental Yeast, Tokyo, Japan) was given ad libitum. NIH-07PLD diet constituents were carbohydrate, crude protein, crude fiber, fat, neutral detergent fiber, ash, fatty acids, amino acids, vitamins, and trace elements with no phytoestrogens. All animals were checked once daily for general behavior and signs of toxicity or a moribund state. Body weights, water and food intakes were measured every week for the first 12 weeks, and thereafter every 4 weeks. During the experiment, the specific signs used to determine when the animals should be euthanized included no response to stimuli or a comatose condition, changes in heart rate and physical appearance, dyspnea or severe breathing problems, hypothermia, prostration, body weight loss, and changes in food and water intakes. If significant body weight loss or the water and food consumption changes were detected, the relevant animals were checked more precisely for other signs of sickness. At euthanization, a systemic macroscopic pathological examination of liver, kidneys, spleen, adrenals, thymus, mammary glands, and uterus was performed. All experimental procedures were conducted with approval and according to the Guidelines of Animal Care and Use Committee of the Osaka City University Medical School.

### 4.4. Short-Term Experiment 1

We performed ovariectomies on 25 female Donryu rats (5 weeks of age) with normal estrous cycles. All animals were checked by vaginal cytology to confirm the absence of estrous cycles. Two weeks after the ovariectomy, for 2 weeks the rats were given (1) PM at doses of 0.03%, 0.3%, and 3%; (2) 0.2% isoflavone aglycone (IA) extract in basal NIH-07PLD diet; or (3) only control diet. Vaginal smears stained with aqueous solution of methylene blue were used to check the estrogenic activity of the test compounds. Animal body weights were measured once a week, and general condition was examined once a day. The weights of uteri were measured at final necropsy to determine estrogenic effects of test compounds. Mammary gland, uterus, liver, kidneys, spleen, adrenals, and thymus were subjected to histopathological analysis.

Donryu rats with a mean body weight of 200 g consumed PM in 15 g diet at doses of 20 (0.03%), 200 (0.3%), 667 (1%), and 2000 (3%) mg/kg b.w./day, considered equal to about 0.2, 2.0, and 20.0 mg/kg b.w./day (10, 50, and 1000 mg/day) intake by women with mean body weight of 50 kg (the acceptable daily intake (ADI) for rats is considered to be 100 times that of humans as the safety factor is 100 (World Health Organization)). As the ADI of PM for humans is 1–2 mg/kg b.w./day, with this extrapolation, the 0.3% PM dose used in our experiment would be accepted as safe for humans (2 mg/kg b.w./day). In addition, the dose for rats could be also extrapolated to a human equivalent dose by the body surface area (BSA) normalization method (mg/m^2^ conversion) [30,31], in which multiplication of the human dose by 6.16 is applied (Km human/Km animal = 37/6). In case of BSA normalization, PM doses of 20, 200, and 2000 mg/kg b.w./day would be equal to 3.2, 32.5, and 325 mg/kg b.w./day intake in humans, and the accepted safe dose of PM is close to 0.03%.

Because of the very strong estrogenic effect of 3% PM observed in this experiment, the largest dose applied in the long-term experiment was changed to 1% (667 mg/kg b.w./day), equal to about 7 mg/kg b.w./day (350 mg/day) for humans, or in the case of BSA normalization, 108 mg/kg b.w./day intake.

### 4.5. Short-Term Experiment 2

DMBA in sesame oil at a dose of 50 mg/kg b.w. was administered by gavage (i.g.) to 20 female Donryu rats (5 weeks of age). Another 5 rats were given an equivalent volume of sesame oil alone (~0.5 mL/rat). PM at doses of 0.03%, 0.3%, and 3% and 0.2% IA were administered in NIH-07PLD diet for 4 weeks starting from the day of DMBA injection. At euthanasia, liver, kidneys, spleen, adrenals, thymus, and uterus were weighed, and samples of mammary glands were fixed in 10% phosphate-buffered formalin for the histopathological and bromodeoxyuridine (BrdU) immunohistochemical examination.

### 4.6. Long-Term Experiment (Experiment 3)

One hundred and seventeen 5-week-old female Donryu rats were divided into seven groups. It is known that soon after weaning, about postnatal day 35, pubertal development of Donryu rats starts. Since the onset of puberty is defined as the age (in days) at which vaginal opening occurs, rats were inspected daily for this purpose. At the commencement of the experiment, 5-week-old rats from PM-treated, IA-treated, and initiation control groups (21 rats/group) were given a single dose of DMBA by i.g. (50 mg/kg b.w.) for initiation of mammary carcinogenesis. On experimental days 7 and 11, ENNG (10 mg/kg b.w.) in PEG was injected via the vagina using a stainless catheter to initiate uterine carcinogenesis. Six rats each in vehicle and 1% PM vehicle groups received sesame oil by i.g. and PEG via the vagina. PM was administered to rats at doses of 0.03%, 0.3%, and 1%, and IA was applied for comparison at a dose of 0.2% in NIH-07PLD basal diet for 36 weeks from the commencement of the experiment. Rats in the vehicle control group received the basal diet. One of the characteristics of Donryu rats is age-related persistent estrus followed by anovulation starting at the age of 5 months, the incidence of which rises until 8 months [32]. Mammary tumor number and volume (cm^3^/rat; the formula: tumor length/2 × (width/2)^2^) were assessed once weekly, and the location of each nodule was recorded. Malignant tumors usually metastasized to the lung, contained abscesses and ulcers, and were of dark color. Histopathologic analysis was performed according to the previously published classification of mammary tumors [33].

All surviving animals were euthanized at week 36 and the test organs—including mammary glands with skin, uterus, vaginas, ovaries, liver, pituitary, adrenals, and thymus—were removed, weighed, and fixed in 10% formalin for histopathological analysis. Twelve specimens were obtained from each uterus in cross-section and proliferative endometrial lesions were classified using the categories reported previously [34] into three degrees (mild, moderate, and severe) of atypical hyperplasia, stromal and adenomatous polyps, and adenocarcinoma.

### 4.7. BrdU Immunohistochemistry

In short-term experiment 2, for evaluation of cellular proliferation, BrdU staining was performed for rat mammary glands by the avidin-biotin-peroxidase complex (ABC) method reported previously [35]. Sections were incubated with mouse monoclonal anti-BrdU antibody (Dako Japan, Kyoto, Japan) at 1:500 dilution. Immunoreactivity was detected using a Vectastain Elite ABC Kit (PK-6102; Vector Laboratories, Burlingame, CA, USA) and 3,3′-diaminobenzidine hydrochloride (Sigma Chemical Co., St. Louis, MO, USA). A negative control was also included with the staining procedure by omitting the primary antibody. At least 3000 mammary epithelial cells’ nuclei were counted in each rat, and labeling indices were calculated as numbers of positive nuclei per 1000 cells.

### 4.8. Blood Hematology and Biochemistry

Blood samples were collected directly from the hearts of all surviving rats at the end of the study after overnight fasting. An automated hematology analyzer (Sysmex XE-2100, Mitsubishi Chemical Visuals, Osaka, Japan) and an automatic analyzer (Olympus AJ-5200, Tokyo, Japan) were employed for hematological and biochemical analyses of blood serum as previously described [36].

### 4.9. Statistical Analysis

Statistical analysis was performed using the StatLight-2000(C) program (Yukms Corp., Tokyo, Japan) or with GraphPad Prism 5 Software Inc. (San Diego, CA, USA). Incidences of histopathological lesions were compared by the Fisher’s exact probability test or the *χ*^2^-test, and the log rank test. The Kaplan–Meier method was employed for assessment of differences in survival. Numerical data for control and experimental groups were statistically compared using the Bartlett’s test. The Dunnett’s multiple comparison test (two-sided) was used in case of homogeneous data, otherwise the Steel’s test (two-sided) was applied [37]. For all data, P values less than 0.05 were considered significant.

## Figures and Tables

**Table 1:** Incidence and multiplicity of neoplastic lesions in the mammary glands and uteri of Donryu rats.

Treatment	No. Rats ^a^	Fibroadenoma		Fibroma	Adenoma			AdCa	
Mammary Glands									
**Incidence (No. Rats (%))**									
DMBA, ENNG	21	20 (95.2)		7 (33.3)	3 (14.3)			1 (4.8)	
DMBA, ENNG → PM, 0.03%	20	19 (95)		8 (40)	2 (10)			6 (30)	
DMBA, ENNG → PM, 0.3%	20	19 (95)		8 (40)	4 (20)			7 (35) *	
DMBA, ENNG → PM 1%	21	20 (95.2)		5 (23.8)	4 (19.1)			6 (28.6)	
DMBA, ENNG → IA, 0.2%	21	18 (85.7)		3 (14.3)	2 (9.5)			6 (28.6)	
Vehicle	5	1 (20)		0	0			0	
Vehicle → PM, 1%	6	1 (16.7)		0	0			0	
**Multiplicity (No./Rat)**									
DMBA, ENNG	21	10.90 ± 4.93 ^d^		0.55 ± 0.89 ^b^	0.15 ± 0.37			0.05 ± 0.22	
DMBA, ENNG → PM, 0.03%	20	9.85 ± 5.39		0.40 ± 0.50	0.10 ± 0.31			0.40 ± 0.68	
DMBA, ENNG → PM, 0.3%	20	8.30 ± 4.93		0.50 ± 0.69	0.20 ± 0.41			0.45 ± 0.69 ^#^	
DMBA, ENNG → PM 1%	21	7.95 ± 4.26		0.29 ± 0.56	0.19 ± 0.40			0.33 ± 0.58	
DMBA, ENNG → IA, 0.2%	21	6.90 ± 5.02 *		0.14 ± 0.36	0.10 ± 0.30			0.43 ± 0.75	
Vehicle	5	0.20 ± 0.45		0	0			0	
Vehicle → PM, 1%	6	0.17 ± 0.41		0	0			0	
**Uterus**		Dilatation	Endometrial HPL				AdCa	Polyps	
			Mild	Moderate	Severe	Total		S	A
**Incidence (No. Rats (%))**									
DMBA, ENNG	21	8 (38.1)	10 (47.6)	5 (23.8)	2 (9.5)	17 (81.0)	1 (4.8)	6 (28.6)	3 (14.3)
DMBA, ENNG → PM, 0.03%	20	13 (65)	16 (80)	4 (20)	1 (5)	17 (85)	3 (15)	5 (25)	2 (10)
DMBA, ENNG → PM, 0.3%	20	19 (95) **	17 (85)	7 (35)	1 (5)	19 (95)	2 (10)	11 (55)	8 (40)
DMBA, ENNG → PM 1%	21	19 (90.5) **	11(52.4)	2 (9.5)	2 (9.53)	12 (57.1)	0 (0)	4 (19.0)	5 (23.8)
DMBA, ENNG → IA, 0.2%	21	19 (90.5) **	16 (76.2)	5 (23.8)	4 (19.0)	20 (95.2)	2 (9.5)	9 (42.9)	9 (42.9)
Vehicle	5	0 (0)	0 (0)	0 (0)	0 (0)	0 (0)	0 (0)	0 (0)	0 (0)
Vehicle → PM, 1%	6	6 (100) *	3 (50.0)	0 (0)	0 (0)	3 (50.0)	0 (0)	0 (0)	0 (0)
**Multiplicity (No./Rat)**									
DMBA, ENNG	21	-	0.67 ± 0.80 ^c^	0.24 ± 0.44	0.14 ± 0.48	1.10 ± 0.64 ^d^	0.05 ± 0.22	0.33 ± 0.58	0.14 ± 0.36
DMBA, ENNG → PM, 0.03%	20	-	1.25 ± 0.91 *	0.24 ± 0.54	0.05 ± 0.22	1.55 ± 1.00	0.15 ± 0.37	0.25 ± 0.44	0.15 ± 0.47
DMBA, ENNG → PM, 0.3%	20	-	1.35 ± 0.81 *	0.35 ± 0.49	0.05 ± 0.22	1.75 ± 0.79 *	0.10 ± 0.31	0.95 ± 1.19	0.65 ± 1.09
DMBA, ENNG → PM 1%	21	-	0.57 ± 0.60	0.10 ± 0.30	0.14 ± 0.48	0.81 ± 0.81	0.00 ± 0.00	0.43 ± 1.33	0.33 ± 0.73
DMBA, ENNG → IA, 0.2%	21	-	1.00 ± 0.71	0.24 ± 0.44	0.19 ± 0.40	1.52 ± 0.60 *	0.10 ± 0.30	0.57 ± 0.75	0.57 ± 0.75 *
Vehicle	5	-	0	0	0	0	0	0	0
Vehicle → PM, 1%	6	-	0.50 ± 0.55	0	0	0.50 ± 0.55	0	0	0

Values are mean ± SD; ^a^ Effective number of rats; * Significantly different from the DMBA, ENNG control group at *p* < 0.05; ^#^ *p* = 0.05; ^b–d^ Significantly different from the Vehicle control group at *p* < 0.05, *p* < 0.01 and *p* < 0.0001; AdCa, adenocarcinoma; HPL, hyperplasia; S, stromal polyp; A, adenomatous polyp.

## Supplementary Materials

The following are available online at www.mdpi.com/2072-6651/8/11/275/s1, Table S1: Final body, relative organ weights and chemicals intakes of Donryu rats in Exp. 3, Table S2: Results of the hematological and blood biochemical analyses.
