# Supplementary material for: Pueraria mirifica Exerts Estrogenic Effects in the Mammary Gland and Uterus and Promotes Mammary Carcinogenesis in Donryu Rats
Source: Toxins (Basel). 2016 Nov 4;8(11):275. doi: 10.3390/toxins8110275 (PMC5127102; doi:10.3390/toxins8110275)
Supplement: Supplementary file 1 [file toxins-08-00275-s001.pdf]

Supplementary Materials: Pueraria *mirifica* Exerts Estrogenic Effects in the Mammary Gland and Uterus and Promotes Mammary Carcinogenesis in Donryu Rats

Anna Kakehashi, Midori Yoshida, Yoshiyuki Tago, Naomi Ishii, Takahiro Okuno, Min Gi and Hideki Wanibuchi

Table S1. Final body, relative organ weights and chemicals intakes of Donryu rats in Exp. 3.

Treament	No. Rats [a]	Final Body Weight (g)	Food Consumption (g/rat/day)	Chemical Intake (mg/rat/day)	Chemical Intake (mg/kg b.w./day)	Total Chemical Intake (g/rat)
DMBA, ENNG	21	375.5 ± 89.4	17.5 ± 3.6	0 ± 0	0 ± 0	0 ± 0
DMBA, ENNG → PM, 0.03%	21	344.1 ± 77.6	17.5 ± 5.8	5 ± 2	21 ± 13	1.4
DMBA, ENNG → PM, 0.3%	21	306.8 ± 82.1 *	19.2 ± 8.4	58 ± 25	263 ± 176	14.9
DMBA, ENNG → PM 1%	21	263.3 ± 49.0 ***	17.9 ± 2.5	179 ± 25	814 ± 171	45.1
DMBA, ENNG → IA, 0.2%	21	323.8 ± 65.7	18.2 ± 4.9	36 ± 10	160 ± 95	9.4
Vehicle	5	372.1 ± 42.9	19.6 ± 3.4	0 ± 0	0 ± 0	0 ± 0
Vehicle → PM, 1%	6	237.3 ± 30.9 #	15.4 ± 3.6	159 ± 23	728 ± 104	40.1

Treatment	No. Rats	Uterus (%)	Liver (%)	Kidneys (%)	Spleen (%)	Thymus (%)	Adrenals (%)
DMBA, ENNG	21	0.33 ± 0.03	4.20 ± 1.92	0.64 ± 0.17	0.51 ± 1.37	0.075 ± 0.091	0.026 ± 0.023
DMBA, ENNG → PM, 0.03%	21	0.41 ± 0.07	3.70 ± 0.79	0.64 ± 0.11	0.34 ± 0.28	0.186 ± 0.144 **	0.022 ± 0.006
DMBA, ENNG → PM, 0.3%	21	0.47 ± 0.06 *	3.84 ± 0.74 *	0.73 ± 0.14	0.22 ± 0.06	0.046 ± 0.021	0.024 ± 0.007
DMBA, ENNG → PM 1%	21	1.35 ± 0.52 *	3.73 ± 0.60 ***	0.79 ± 0.14 ***	0.37 ± 0.48	0.048 ± 0.018	0.031 ± 0.016
DMBA, ENNG → IA, 0.2%	21	0.36 ± 0.04	3.82 ± 1.51	0.66 ± 0.10	0.35 ± 0.31	0.054 ± 0.019	0.023 ± 0.005
Vehicle	5	0.27 ± 0.02	2.66 ± 0.26	0.58 ± 0.05	0.17 ± 0.03	0.063 ± 0.022	0.016 ± 0.003
Vehicle → PM, 1%	6	0.99 ± 0.71	3.20 ± 0.16	0.81 ± 0.07 #	0.19 ± 0.03	0.065 ± 0.014	0.022 ± 0.003 ##

[a] Effective number of rats, * $p < 0.05$; ** $p < 0.01$; *** $p < 0.001$ vs. DMBA, ENNG group; # $p < 0.05$; ## $p < 0.01$ vs. vehicle control group.

Table S2. Results of the hematological and blood biochemical analyses.

Test Compound	DMBA, ENNG					Vehicle	
	PM				IA	Vehicle	PM
Dose of test compound (%)	0	0.03	0.3	1	0.2	0	1
No. of animals examined	17	15	13	19	15	5	6
WBC (/μL)	4550 ± 1777	6479 ± 8488	4525 ± 2235	4141 ± 2463	7547 ± 12,949	3600 ± 1706	3320 ± 996
RBC ($\times10^4$/μL)	593.0 ± 95.6 #	589.4 ± 112.3	635.1 ± 125.7	612.7 ± 113.8	625.9 ± 185.1	770.4 ± 32.6	728.6 ± 31.9
Hb (g/dL)	11.7 ± 1.6 #	11.6 ± 2.6	11.94 ± 2.0	11.6 ± 2.0	11.8 ± 3.4	14.1 ± 0.6	13.9 ± 1.1
Ht (%)	38.0 ± 4.7 #	35.7 ± 7.3	37.9 ± 5.8	36.6 ± 6.0	37.6 ± 10.1	44.7 ± 0.9	42.1 ± 1.8 #
MCV (fL)	64.6 ± 4.2	60.6 ± 5.0	60.4 ± 4.6	60.1 ± 3.3	62.3 ± 8.2	58.2 ± 1.8	57.8 ± 0.8
MCH (pg)	19.9 ± 1.0	19.9 ± 5.7	18.9 ± 1.0	19.0 ± 0.8	19.3 ± 2.4	18.4 ± 0.8	19.1 ± 1.1
MCHC (g/dL)	30.8 ± 0.8	32.8 ± 8.0	31.4 ± 1.2	31.7 ± 0.9	31.1 ± 1.9	31.6 ± 0.9	32.9 ± 2.0
Platelets ($\times10^{10}$/L)	76.7 ± 13.0 ##	57.4 ± 24.2 *	39.1 ± 31.9 **	54.2 ± 25.5 **	56.2 ± 28.5	38.5 ± 20.0	56.4 ± 15.8
Neutrophils ($\times10^3$/L)	32.2 ± 17.0	42.1 ± 23.9	25.8 ± 15.1	34.3 ± 14.4	31.9 ± 15.7	30.0 ± 5.6	42.8 ± 8.1 #
Band neutrophils ($\times10^3$/L)	1.4 ± 0.6	1.1 ± 0.4	1.2 ± 0.4	1.1 ± 0.2	1.4 ± 0.9	1.0 ± 0.0	1.0 ± 0.0
Eosinophils ($\times10^3$/L)	1.2 ± 1.2	0.9 ± 1.2	0.7 ± 1.0	0.7 ± 1.6	0.7 ± 1.3	1.0 ± 0.7	0.6 ± 0.5
Basophils ($\times10^3$/L)	0.0 ± 0.0	0.0 ± 0.0	0.0 ± 0.0	0.0 ± 0.0	0.0 ± 0.0	0.0 ± 0.0	0.0 ± 0.0
Monocytes ($\times10^3$/L)	2.9 ± 1.4	2.4 ± 1.5	3.5 ± 1.3	3.9 ± 2.3	2.8 ± 1.8	3.0 ± 1.6	3.8 ± 1.5
Lymphocytes ($\times10^3$/L)	62.3 ± 16.7	53.5 ± 24.0	68.8 ± 15.8	60.1 ± 14.0	62.7 ± 16.2	65.0 ± 6.7	51.6 ± 9.8 #
T-protein (g/dL)	7.3 ± 1.0	6.7 ± 0.6	7.1 ± 0.5	6.8 ± 0.8	7.2 ± 0.4	7.5 ± 0.4	7.6 ± 0.5
Albumin (g/dL)	5.4 ± 0.5	4.8 ± 0.8	5.2 ± 0.6	4.9 ± 0.8	5.3 ± 0.5	5.1 ± 0.4	5.4 ± 0.4
A/G ratio	3.2 ± 0.9	2.6 ± 0.8	3.0 ± 0.9	2.6 ± 0.7	3.0 ± 0.7	2.1 ± 0.4	2.6 ± 0.5
T-BiL (mg/dL)	0.10 ± 0.00	0.10 ± 0.00	0.10 ± 0.00	0.11 ± 0.02	0.11 ± 0.03	0.14 ± 0.1	0.10 ± 0.00
AST (IU/L)	428.1 ± 216.9	425.1 ± 528.6	378.2 ± 295.2	263.7 ± 132.6 *	375.2 ± 296.6	467.0 ± 105.7	223.2 ± 19.4 ##
ALT (IU/L)	62.7 ± 39.8	62.8 ± 77.6	87.4 ± 84.0	55.6 ± 28.8	88.8 ± 89.1	96.2 ± 41.1	55.7 ± 12.0 #
ALP (IU/L)	62.0 ± 34.0	147.7 ± 253.6	73.6 ± 18.3	99.7 ± 58.6 *	77.5 ± 38.3	163.9 ± 98.1	125.3 ± 51.0
γ-GTP (IU/L)	2.1 ± 3.1	3.0 ± 2.9	2.5 ± 1.6	5.5 ± 11.7 **	3.9 ± 2.9 **	1.0 ± 0.0	2.2 ± 1.0 #
T-Cholesterol (mg/dL)	141.5 ± 35.4 #	123.5 ± 48.2	123.0 ± 30.0	105.3 ± 27.9 *	130.0 ± 36.9	191.6 ± 39.0	116.7 ± 31.8 ##
TG (mg/dL)	90.6 ± 51.5 #	76.7 ± 44.7	65.5 ± 60.2	68.1 ± 46.3	111.9 ± 169.2	47.4 ± 19.2	39.7 ± 28.4
BUN (mg/dL)	22.3 ± 4.1 #	25.1 ± 7.2	24.2 ± 4.0	27.1 ± 9.9	22.0 ± 7.0	17.2 ± 2.2	23.7 ± 4.4 #
Creatinine (mg/dL)	0.31 ± 0.07	0.33 ± 0.05	0.35 ± 0.06	0.29 ± 0.08	0.29 ± 0.04	0.37 ± 0.05	0.36 ± 0.03
Na (mEq/L)	143.9 ± 1.4	143.9 ± 1.3	143.0 ± 2.7	143.4 ± 1.8	143.4 ± 1.8	143.6 ± 1.9	144.7 ± 1.6
K (mEq/L)	5.0 ± 0.9	5.1 ± 0.5	5.6 ± 3.7	4.8 ± 0.9	4.9 ± 0.7	4.8 ± 0.3	4.6 ± 0.4
Cl (mEq/L)	100.1 ± 1.7	100.5 ± 1.7	99.8 ± 1.6	100.2 ± 2.0	100.5 ± 2.1	98.4 ± 1.9	99.2 ± 1.8
Ca (mEq/L)	9.9 ± 0.4	9.7 ± 0.4	9.7 ± 0.4	9.4 ± 0.4 ***	9.6 ± 0.3	10.2 ± 0.5	9.7 ± 0.4
IP (mEq/L)	6.8 ± 1.6	7.2 ± 1.4	6.3 ± 2.5	5.9 ± 1.2	5.5 ± 1.7	7.9 ± 3.2	6.4 ± 1.1

Values are means ± SD; * $p < 0.05$;** $p < 0.01$; *** $p < 0.001$ vs. DMBA, ENNG initiation control group; # $p < 0.05$; ## $p < 0.01$ vs. vehicle control group; TG, triglycerides; T-Bil, T-bilirubin; IP, inorganic phosphorus.
